# Determining Vitamin D Status: A Comparison between Commercially Available Assays

**DOI:** 10.1371/journal.pone.0011555

**Published:** 2010-07-13

**Authors:** Greta Snellman, Håkan Melhus, Rolf Gedeborg, Liisa Byberg, Lars Berglund, Lisa Wernroth, Karl Michaëlsson

**Affiliations:** 1 Section of Orthopaedics, Department of Surgical Sciences, University Hospital, Uppsala, Sweden; 2 Section of Clinical Pharmacology, Department of Medical Sciences, University Hospital, Uppsala, Sweden; 3 Uppsala Clinical Research Centre, University Hospital, Uppsala, Sweden; 4 Section of Anaesthesiology and Intensive Care, Department of Surgical Sciences, University Hospital, Uppsala, Sweden; University of Michigan, Canada

## Abstract

**Background:**

Vitamin D is not only important for bone health but can also affect the development of several non-bone diseases. The definition of vitamin D insufficiency by serum levels of 25-hydroxyvitamin D depends on the clinical outcome but might also be a consequence of analytical methods used for the definition. Although numerous 25-hydroxyvitamin D assays are available, their comparability is uncertain. We therefore aim to investigate the precision, accuracy and clinical consequences of differences in performance between three common commercially available assays.

**Methodology/Principal Findings:**

Serum 25-hydroxyvitamin D levels from 204 twins from the Swedish Twin Registry were determined with high-pressure liquid chromatography-atmospheric pressure chemical ionization-mass spectrometry (HPLC-APCI-MS), a radioimmunoassay (RIA) and a chemiluminescent immunoassay (CLIA). High inter-assay disagreement was found. Mean 25-hydroxyvitamin D levels were highest for the HPLC-APCI-MS technique (85 nmol/L, 95% CI 81–89), intermediate for RIA (70 nmol/L, 95% CI 66–74) and lowest with CLIA (60 nmol/L, 95% CI 56–64). Using a 50-nmol/L cut-off, 8% of the subjects were insufficient using HPLC-APCI-MS, 22% with RIA and 43% by CLIA. Because of the heritable component of 25-hydroxyvitamin D status, the accuracy of each method could indirectly be assessed by comparison of within-twin pair correlations. The strongest correlation was found for HPLC-APCI-MS (r = 0.7), intermediate for RIA (r = 0.5) and lowest for CLIA (r = 0.4). Regression analyses between the methods revealed a non-uniform variance (p<0.0001) depending on level of 25-hydroxyvitamin D.

**Conclusions/Significance:**

There are substantial inter-assay differences in performance. The most valid method was HPLC-APCI-MS. Calibration between 25-hydroxyvitamin D assays is intricate.

## Introduction

Vitamin D deficiency is not only associated with osteoporosis and osteomalacia [1], [2] but can also contribute to decreased muscle strength [3], cancers [4], cardiovascular disease [5], type 1 diabetes mellitus [6] and overall mortality [7]. Defined risk groups are elderly, dark-skinned and obese, as well as inhabitants in northern latitudes where UV B radiation is undetectable during winter [8], [9], [10], [11]. These findings have increased the need for determining vitamin D status in a reliable way.

Vitamin D exists in two forms, namely ergocalciferol (vitamin D_2_), and cholecalciferol (vitamin D_3_). Vitamin D status is assessed by measuring serum levels of 25-hydroxyvitamin D. Fatty fish and dairy products are the main dietary sources of vitamin D_3_ [12], [13]. The most important source of circulating 25-hydroxyvitamin D_3_ is, with sufficient solar exposure, the endogenous dermal production of pre-vitamin D_3_ after exposure to UV B radiation [8]. The less dominant serum 25-hydroxyvitamin D_2_ is mainly derived from plant foods and in some countries from supplements.

There are many commercially available 25-hydroxyvitamin D assays used for determination of vitamin D status. These include high-pressure liquid chromatography (HPLC) and mass spectrometry (MS) [14], radioimmunoassays (RIA), enzyme immunoassays (EIA), competitive protein binding assays (CPBA), automated chemiluminescence protein-binding assays (CLPBA) and chemiluminescent immunoassays (CLIA). All these assays are used in both clinical and research settings but it is not widely appreciated that 25-hydroxyvitamin D assays may yield discrepant results. Inter-assay and laboratory disagreement could contribute to uncertainty when comparing results from studies investigating the prevalence or clinical consequence of vitamin D insufficiency. Indeed, several studies including reports from DEQAS, an organization who aim to ensure the analytical reliability of 25-hydroxyvitamin D assays [15], have indicated high variability between different assays as well as inter-laboratory disagreement, but these studies have been limited by few participants, a non-population based setting, only partially overlapping analyses of the samples included, and consensus opinion regarding accuracy rather than an unbiased comparator [16], [17], [18], [19], [20], [21], [22].

25-hydroxyvitamin D levels are partially genetically determined [23], [24], [25]. Higher twin resemblance in serum 25-hydroxyvitamin D values indicates enhanced performance of the assay. This fact, thus, enables that the within-pair correlation to be used as an unbiased proxy measure of accuracy. Determination of accuracy has not been possible in previous validation studies [16], [17], [18], [19], [20], [21]. In these studies, it has also not been evaluated if the differences in assay results have been uniform or have been dependent on serum 25-hydroxyvitamin D level. Cross-calibration between assays is dependent on the pattern of assay differences. We therefore aimed in a twin study to compare the differences in performance between three common commercially available methods for 25-hydroxyvitamin D analysis with different methodological principles: a combined HPLC-MS method, a RIA and a CLIA assay.

## Materials and Methods

### Ethics Statement

The study protocol was approved by the Ethics Committee of Uppsala University and all participants gave written informed consent to participate in the study and to donate blood samples.

### Subjects

Subjects were recruited from The Swedish Twin Registry. All intact like-sexed twin pairs, born 1965 or earlier and living in the county of Uppsala were invited to participate. Uppsala County is located in central Sweden at northern latitude 60°. Totally, 172 twin pairs were found eligible and invited to participate. Of these, 102 twin pairs, i.e. 204 subjects, accepted to participate in the study. No subjects were excluded. Zygosity information in the Swedish twin registry has a high validity [26]. The study included 59 female and 43 male Caucasian twin pairs with an age range between 39 and 85 years. Because of low UV B radiation, vitamin D cannot be synthesized in the skin between late autumn through April at high latitudes [27]. We therefore defined winter as November throughout April and summer as May throughout October. The serum samples were collected during the winter season for 28 twin pairs and during the summer season for 74 pairs. When possible, both members of each pair were examined within the same week to take the individual seasonal variation in vitamin D levels into account. Sixty-one pairs were examined the same day. The median within pair difference in days between the examinations among the remainder of the pairs was 6 days (inter quartile range 2 to 9 days), with a maximum of 17 days. Seven twins reported use of vitamin D supplements.

The study protocol was approved by the local ethical committee of Uppsala and all participants gave written informed consent to participate in the study and to donate blood samples.

### Biochemical analyses

Venous blood samples were collected after a 12-h overnight fasting, protected from light, centrifuged and stored at −80°C until analysis. All samples were analyzed in three laboratories using three techniques (described in detail below).

#### High-pressure liquid chromatography (HPLC) - atmospheric pressure chemical ionisation (APCI) - mass spectrometry (MS)

Determination of 25-hydroxyvitamin D_2_ and D_3_ in plasma with HPLC-APCI-MS was done at Vitas, Oslo, Norway. Deuterium labelled 25-hydroxyvitamin D_2_ and D_3_ were used for internal standards. One hundred and fifty µL of human plasma were diluted with 450 µL 2-propanol containing BHT (butylhydroxytoluene) as an antioxidant. After thorough mixing (15 min) and centrifugation (10 min, 4000 g at 10°C), an aliquot of 35 µL was injected from the supernatant into the HPLC system. HPLC was performed with a HP 1100 liquid chromatograph (Agilent Technologies, Palo Alto CA, USA) interfaced by atmospheric pressure chemical ionization (APCI) to a HP mass spectrometric detector (MS) operated in single-ion monitoring mode (SIM). 25-hydroxyvitamin D_2_ and D_3_ were separated on a 4.6 mm ×50 mm reversed phase column with 1.8 µM particles. The column temperature was 80°C. A two-point calibration curve was made from analysis of albumin solution enriched with known vitamin D concentration. Recovery is 95%; the method is linear from 5–400 nmol/L and the limit of detection is 1–4 nmol/L. The Coefficients of Variation (CV) for inter-assay analyses are 7.6% at 25-hydroxyvitamin D of 47.8 nmol/L and 6.9% at 25-hydroxyvitamin D of 83.0 nM. The assay is accredited by the Vitamin D External Quality Assessment Scheme (DEQAS) [15].

#### Radioimmunoassay (RIA)

25-hydroxyvitamin D_2_ and D_3_ in serum were measured at a research laboratory in Uppsala using Gamma-B 25-hydroxyvitamin D RIA (IDS, Boldon, UK). The CV for inter-assay analyses is 7.9%. Sensitivity, defined as the concentration corresponding to the mean minus 2 standard deviations of 10 replicates of the zero calibrator, is <3 nmol/L. To ascertain analytic quality all standards, controls and samples were analyzed in duplicate and all duplicates with a coefficient of variation >10% were reanalyzed. The control samples provided by the manufacturer were within the recommended range.

#### Chemiluminescent immunoassay (CLIA)

25-hydroxyvitamin D_2_ and D_3_ in serum were measured as a standard procedure at the department of Clinical Chemistry at Uppsala University Hospital. The LIAISON® 25-hydroxyvitamin D Assay (DiaSorin) uses chemiluminescent immunoassay technology. Specific antibody to vitamin D is used for coating magnetic particles (solid phase) and vitamin D is linked to an isoluminol derivative. During the incubation, 25-hydroxyvitamin D is dissociated from its binding protein and competes with labelled vitamin D for binding sites on the antibody. After the incubation, the unbound material is removed with a wash cycle. Subsequently, the starter reagents are added and a flash chemiluminescent reaction is initiated. The light signal is measured by a photomultiplier as relative light units and is inversely proportional to the concentration of 25-hydroxyvitamin D present in samples. CV for inter-assay analyses is 18.4% at a 25-hydroxyvitamin D level of 39.5 nmol/L and 11.7% at 121.25 nmol/L. The quality of the method is evaluated using the Vitamin D External Quality Assessment Scheme (DEQAS) [15] that is, based on blinded samples with varying concentrations of 25(OH)D, sent out as a within- and between-method comparison to over 500 participating laboratories, the assay results agree within ±30% of All Laboratory Trimmed Mean (ALTM) [15].

### Statistical analysis

The 25-hydroxyvitamin D values were normally distributed with Shapiro-Wilk's w value greater than 0.95. Mean serum 25-hydroxyvitamin D values with 95% confidence intervals (CI) for each assay results were calculated including stratification by season. The cumulative proportion of twins at each serum level by method was plotted in order to compare the proportion of twins with values below each 25-hydroxyvitamin D level, specifically the 50 nmol/L insufficiency level proposed by expert opinion [28], [29]. To estimate the accuracy of each method, resemblance in results within twin pairs was calculated by intraclass correlation coefficients (ICCs). The sample coefficient of variation (SCV) was calculated by dividing the standard deviation for each method by its mean value. Taking into account the twinship dependence, bootstrap-estimated 95% confidence intervals of ICCs and SCVs, and p values for assay differences, were obtained by re-sampling the total sample size 10,000 times. Additionally, results of assays were compared using Bland-Altman plots [30], [31]. The difference in performance by level of serum 25-hydroxyvitamin D was formally tested by linear regression analysis of the difference in absolute values between two methods regressed against the mean of the method results of the two analyses, also taking into account the twin-ship dependence and zygosity. A p-value <0.05 was considered significant.

## Results

**Figure 1:**
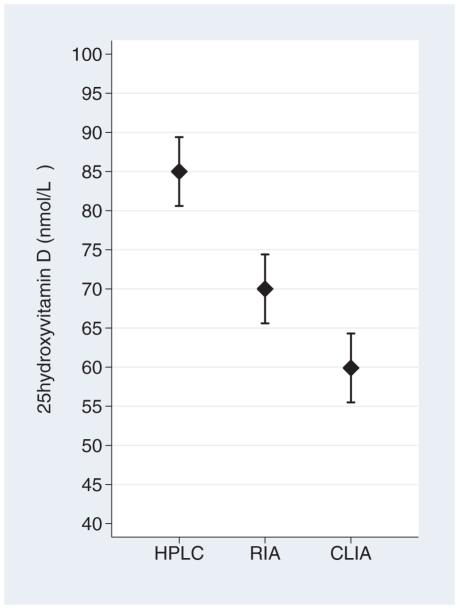
Mean serum 25-hydroxyvitamin D by assay. The error bars indicate 95% confidence intervals.

**Figure 2:**
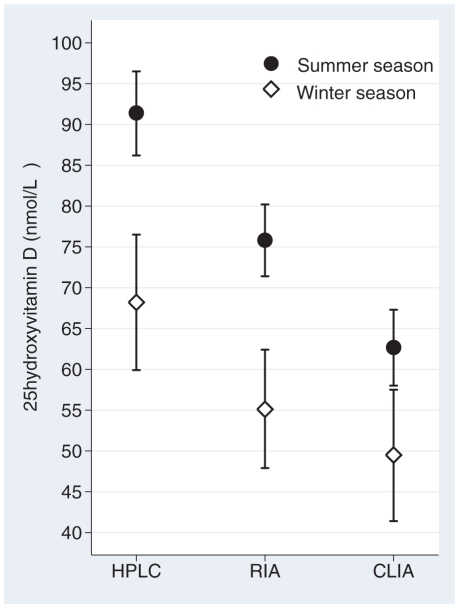
Seasonal differences in 25-hydroxyvitamin D levels for the HPLC-APCI-MS, RIA and CLIA assays. The error bars indicate 95% confidence intervals.

Our results reveal low inter-assay agreement. Mean 25-hydroxyvitamin D values and basic characteristics of the twins are presented in Table 1. HPLC-APCI-MS measured a mean 25-hydroxyvitamin D level of 85 nmol/L, RIA 70 nmol/L and CLIA 60 nmol/L, p for difference between assays <0.0001 (Figure 1). 25-hydroxyvitamin D_2_ was detectable in only 20% (n = 40) of our subjects using the HPLC-APCI-MS assay. The mean level among these 40 twins was 8 nmol/L, and they contributed to only 1.5 nmol/L of the mean 25-hydroxyvitamin D level among all 204 twins. Demonstrated in Figure 2, the serum 25-hydroxyvitamin D levels were significantly higher with all methods during the summer compared to the winter season. The greatest inter-seasonal difference, 23 nmol/L (95% CI 13–33), is presented by the HPLC-APCI-MS assay.

**Table 1:** Mean 25-hydroxyvitamin D (25(OH)D)) values and characteristics of the twins as a function of season.

	Total (n = 204)	
	Mean (SD)	Range
**S-25(OH)D2+3 HPLC-APCI-MS (nmol/L)**	85.0 (27.4)	21.4−181.3
**S-25(OH)D3 HPLC-APCI-MS (nmol/L)**	83.3 (27.4)	21.4−174.4
**S-25(OH)D2 HPLC-APCI-MS (nmol/L)** *	7.7 (2.8)	4.6−13.9
**S-25(OH)D RIA (nmol/L)**	70 (24.0)	26.1−156.5
**S-25(OH)D CLIA (nmol/L)**	59.9 (26.1)	10.0−172.2
**P-PTH (pmol/L)**	1.9 (0.9)	0.4−5.3
**Age (years)**	57.5 (9.7)	37.8−84.5
**Weight (kg)**	74 (12.2)	47.9−122.6
**Height (cm)**	170.5 (9.7)	149.0−194.5
**Body mass index (kg/m^2^)**	25.4 (3.2)	18.9−39.6

Mean 25-hydroxyvitamin D levels are adjusted for the twin-ship dependence.

*Results are based on the 40 (20%) participants who had measurable levels of 25-hydroxyvitamin D_2_.

**Figure 3:**
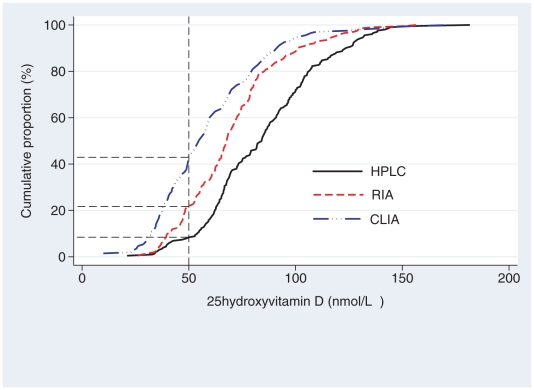
Cumulative proportion of the subjects who are classified as insufficient using a 50 nmol/L cut-off. HPLC-APCI-MS 8%, RIA 22%, CLIA 43%.

There were considerable differences between the methods in the proportion of participants classified as vitamin D insufficient. Using a 50-nmol/L cut-off, only 8% of our subjects were classified as vitamin D insufficient with the HPLC-APCI-MS method, 22% using RIA and 43% with the CLIA method (Figure 3).

As measures of assay accuracy, intraclass correlation coefficients (ICC) for within twin pair similarity in 25-hydroxyvitamin D levels, and 95% CI are presented in Table 2. HPLC-APCI-MS had a significantly higher ICC relative to both RIA and CLIA, and RIA had had a higher value than CLIA. The precision of the assays was determined by sample coefficient of variation (SCV). HPLC-APCI-MS had a SCV of 32%, RIA 34% and CLIA 44% (Table 3).

**Table 2:** Intraclass correlation coefficients (ICC) for serum 25-hydroxyvitamin D levels between twins in a pair with 95% confidence intervals.

Variable	ICC (95% CI)	P-value	
**HPLC-APCI-MS**	0.66 (0.54−0.76)	ref	NA
**RIA**	0.54 (0.39−0.66)	0.004	ref
**CLIA**	0.40 (0.22−0.55)	<0.001	<0.001

The p-values correspond to a test of equality of the observed correlations.

**Table 3:** Sample coefficient of variation (SCV) values with 95% confidence intervals.

Variable	SCV% (95% CI)*	P-value	
**HPLC-APCI-MS**	32.3 (28.5−36.0)	ref	NA
**RIA**	34.2 (29.8−38.3)	0.243	ref
**CLIA**	43.5 (37.7−48.9)	<0.001	0.001

*Adjusted for twin-ship dependence.

**Figure 4:**
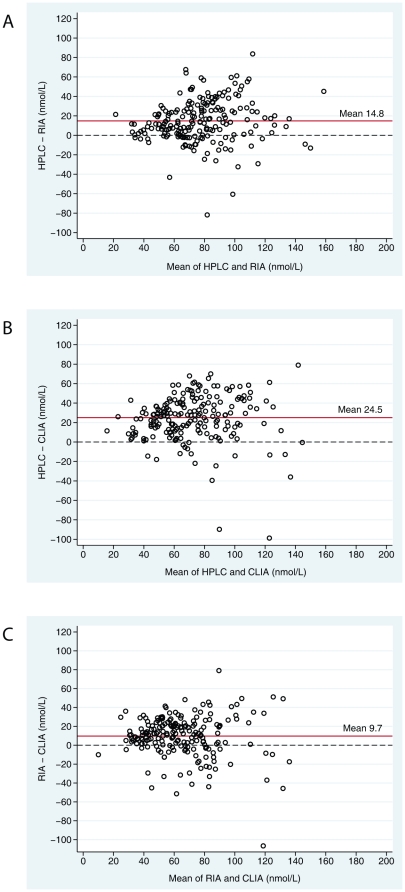
Bland Altman plots for the difference in 25-hydroxyvitamin D level between the assays. HPLC-APCI-MS vs. RIA (panel A), HPLC-APCI-MS vs. CLIA (panel B) and RIA vs. CLIA (panel C). Each circle represents one twin.

According to the Bland-Altman plots, RIA and CLIA had a non-proportional bias relative to HPLC-APCI-MS (Figure 4, panel A–C). Both positive and negative bias became more accentuated with increasing 25-hydroxyvitamin D value, i.e. the inter-assay disagreement increases with an increasing serum level of 25-hydroxyvitamin D. To formally test that there was a non-uniform variability at different serum levels of 25-hydroxyvitamin D between the methods we used linear regression to analyze the relation between absolute differences in serum values between the methods against the mean of the two values (Table 4). Both parameter estimates were positive and highly statistically significantly different from zero, and accordingly, the differences in variation between the methods were higher at increasing levels of serum 25-hydroxyvitamin D.

**Table 4:** Linear regression analyses for the relationship between the absolute differences85 in serum values between the methods against the mean of the two values.

Variable	Slope	95% CI	P-value
**CLIA vs** **HPLC-APCI-MS**	0.19	0.09−0.29	<0.0001
**RIA vs** **HPLC-APCI-MS**	0.22	0.12−0.31	<0.0001

*Adjusted for twin-ship dependence.

## Discussion

We observed high variability between the HPLC-APCI-MS, RIA and CLIA assay results, emphasizing that a gold standard for the 25-hydroxyvitamin D assay is needed to establish consensus on the required level for sufficient vitamin D status. Highest accuracy was found with the HPLC-APCI-MS and lowest with the CLIA method.

Previous studies support our findings, reporting variability between different assays as well as inter-laboratory differences using the same assay [16], [17], [18], [19], [21]. Lips et al [21] analyzed samples from a selected population of vitamin D supplement users (n = 104) with three different methods. The mean 25-hydroxyvitamin D level was 80% higher when using a competitive protein-binding assay as compared with HPLC while intermediate values were found with a RIA assay. The accuracy of the methods was not possible to evaluate, however. Binkley et al reported 18% and 90% insufficiency proportions in two similar populations (n = 20 and 42, respectively) using two RIA assays. IDS and DiaSorin-RIA as well as a Nichols Advantage automated protein binding assay detected less than 50% of the changes in 25-hydroxyvitamin D_2_ detected by HPLC according to Glendenning et al [19].

HPLC can discriminate 25-hydroxyvitamin D_2_ and D_3_ metabolites, whereas our RIA and CLIA method measure total 25-hydroxyvitamin D levels, i.e., the sum of 25-hydroxyvitamin D_2_ and D_3_ metabolites. In some countries, including the USA, vitamin D_2_ has been the only form of vitamin D available for prescription, even though both vitamin D_2_ and vitamin D_3_ are used as non-prescribed supplements, while in Europe vitamin D_3_ is dominating [32]. HPLC could therefore have an advantage when evaluating the effect of supplementation with D_2_. Nevertheless, this is of minor importance in our setting since only 20% of our participants have measurable 25-hydroxyvitamin D_2_ values, contributing on average to only 2% higher total 25-hydroxyvitamin D levels in the cohort. Similar low values were found by Högström et al [33] in young, healthy Swedish men. Thus, in European countries it may not be of major clinical importance that some assays underestimate D_2_ (although not relevant to our study) or cannot separate between D_2_ and D_3_.

There is no well defined and international accepted definition of optimal serum 25-hydroxyvitamin D value for bone and nutritional health [28], [29], but low plasma 25-hydroxyvitamin D and secondary hyperparathyroidism are the biochemical hallmarks for insufficient vitamin D status [2]. A common definition of vitamin D insufficiency is 25-hydroxyvitamin D <50 nmol/L although many authors suggest that clinicians should aim at higher levels [28], [29], [34]. Our great inter-assay differences in insufficiency proportion, as well as in accuracy and in precision indicate that it is questionable to rely on immunoassays when determining whether a patient is insufficient. Some assays may be too imprecise for both clinical and research use. Assay-specific decision limits to define appropriate thresholds for insufficiency have been suggested as a solution [19] but that will be cumbersome for the clinician. Furthermore, our results suggest that a simple reliable calibration between assays cannot be accomplished.

Assay disagreement and inter-laboratory variation naturally have important clinical implications. It may hamper comparison of studies from diverse populations and countries, exemplified by Lips [21]. This may be the reason why there is inconsistent evidence regarding the degree of association between vitamin D status, bone mineral density and the risk of low energy fractures [35].

Because the HPLC-APCI-MS method provides the most prominent difference between summer and winter levels of 25-hydroxyvitamin D, the lowest SCV value and the best twin resemblance in serum levels, our conclusion is that HPLC-APCI-MS is a more accurate and reliable method than both RIA and CLIA.

The advantages of our study are the comparable large sample size, the population-based design, that our participants comprised of twins and that all the samples are analyzed with all three assays. One limitation is that other available methods for 25-hydroxyvitamin D measurement were not evaluated. Moreover, with analyses performed at three laboratories, such a design could limit the ability to separate assay-specific from laboratory-specific bias. Differences between laboratories may be caused by unfamiliarity with the analytical method, but all laboratories in our study were experienced with the method used at that site and all methods are used for clinical decision making. Moreover, Vitas is an approved R&D institution in the SkatteFUNN scheme held by the Norwegian Research Council and is the national reference laboratory for vitamin D analysis. The department of Clinical Chemistry at the Uppsala University Hospital is accredited by the Swedish Board for Accreditation and Conformity Assessment (SWEDAC). SWEDAC must in its turn fulfil certain requirements that are set out in ISO/IEC 17011. Compliance is confirmed by the international assessments that are performed within the framework of the European Accreditation cooperation (EA).

We conclude that a single threshold value to define an optimal 25-hydroxyvitamin D level is presently impossible to determine because of differences in assay results.
