# Correction: Determining Vitamin D Status: A Comparison between Commercially Available Assays

**DOI:** 10.1371/annotation/23307aa4-726e-4f11-86c0-8a292be33517

**Published:** 2010-09-29

**Authors:** Greta Snellman, Håkan Melhus, Rolf Gedeborg, Liisa Byberg, Lars Berglund, Lisa Wernroth, Karl Michaëlsson

University Hospital, Uppsala, Sweden is incorrectly used in the affiliations. It should be replaced with Uppsala University, Uppsala, Sweden.

